# The Association of Myo-Inositol and Selenium Contrasts Cadmium-Induced Thyroid C Cell Hyperplasia and Hypertrophy in Mice

**DOI:** 10.3389/fendo.2021.608697

**Published:** 2021-02-25

**Authors:** Salvatore Benvenga, Antonio Micali, Antonio Ieni, Alessandro Antonelli, Poupak Fallahi, Giovanni Pallio, Natasha Irrera, Francesco Squadrito, Giacomo Picciolo, Domenico Puzzolo, Letteria Minutoli

**Affiliations:** ^1^ Department of Clinical and Experimental Medicine, University of Messina, Messina, Italy; ^2^ Department of Biomedical and Dental Sciences and Morphofunctional Imaging, University of Messina, Messina, Italy; ^3^ Department of Human Pathology, University of Messina, Messina, Italy; ^4^ Department of Clinical and Experimental Medicine, University of Pisa, Pisa, Italy; ^5^ Department of Translational Research and New Technologies in Medicine and Surgery, University of Pisa, Pisa, Italy

**Keywords:** cadmium, myo-inositol, seleno-L-methionine, resveratrol, thyroid, calcitonin, C cells

## Abstract

Previous studies have demonstrated that, in addition to inducing structural changes in thyroid follicles, cadmium (Cd) increased the number of C cells. We examined the effects of myo-inositol (MI), seleno-L-methionine (Se), MI + Se, and resveratrol on C cells of mice exposed to cadmium chloride (Cd Cl2), as no data are currently available on the possible protective effects of these molecules. In contrast, we have previously shown this protective effect against CdCl2 on the thyroid follicles of mice. Ninety-eight C57 BL/6J adult male mice were divided into 14 groups of seven mice each: (i) 0.9% NaCl (vehicle; 1 ml/kg/day i.p.); (ii) Se (0.2 mg/kg/day per os); (iii) Se (0.4 mg/kg/day per os); (iv) MI (360 mg/kg/day per os); (v) Se (0.2 mg/kg/day) + MI; (vi) Se (0.4 mg/kg/day) + MI; (vii) resveratrol (20 mg/kg); (viii) CdCl2 (2 mg/kg/day i.p.) + vehicle; (ix) CdCl2 + Se (0.2 mg/kg/day); (x) CdCl2 + Se (0.4 mg/kg/day); (xi) CdCl2 + MI; (xii) CdCl2 + Se (0.2 mg/kg/day) + MI; (xiii) CdCl2 + Se (0.4 mg/kg/day) + MI; (xiv) CdCl2 + resveratrol (20 mg/kg). After 14 days, thyroids were processed for histological, immunohistochemical, and morphometric evaluation. Compared to vehicle, Cd significantly decreased follicle mean diameter, increased CT-positive cells number, area and cytoplasmic density, and caused the disappearance of TUNEL-positive C cells, namely, the disappearance of C cells undergoing apoptosis. Se at either 0.2 or 0.4 mg/kg/day failed to significantly increase follicular mean diameter, mildly decreased CT-positive cells number, area and cytoplasmic density, and was ineffective on TUNEL-positive C cells. Instead, MI alone increased significantly follicular mean diameter and TUNEL-positive cells number, and decreased significantly CT-positive cells number, area and cytoplasmic density. MI + Se 0.2 mg/kg/day or MI + Se 0.4 mg/kg/day administration improved all five indices more markedly. Indeed, follicular mean diameter and TUNEL-positive cells number increased significantly, while CT-positive cells number, area and cytoplasmic density decreased significantly. Thus, all five indices overlapped those observed in vehicle-treated mice. Resveratrol improved significantly all the considered parameters, with a magnitude comparable to that of MI alone. In conclusion, the association Myo + Se is effective in protecting the mouse thyroid from the Cd-induced hyperplasia and hypertrophy of C cells. This benefit adds to that exerted by Myo + Se on thyrocytes and testis.

## Introduction

The thyroid gland is formed by two main components: the follicular cells and the parafollicular C cells. The follicular apparatus derives from the ventral floor of the anterior pharyngeal endoderm, and is formed by about 3 million spherical structures limited by a single, continuous layer of epithelial cells, the thyrocytes. The parafollicular C cells derive from the endodermal epithelial cells of the fourth pharyngeal pouch (1, 2) and not, as previously believed, from the neural crest cells (3). C cells comprise a small amount of the total cellular population of the thyroid, and are placed between the basement membrane and the thyrocytes, isolated or arranged in solid cell nests (4).

C cells are round or polygonal, with a central nucleus and clear cytoplasm. Because they are difficult to detect with the hematoxylin-eosin (HE) stain (5), the best approach to identify C cells is immunohistochemistry with an antibody against calcitonin (CT), which is the main content of their secretory granules (3). As to location of C cells within the human thyroid gland, they are mainly found at the junction between the upper and the middle part of each lobe (6). However, in rodents C cells abound in the middle and/or caudal portions of the thyroid lobes (C cells region), and are absent in the peripheral part of lobes (7–9).

The thyroid is one of the targets of the “endocrine-disrupting chemicals” (EDCs)  (10–17). One EDC is cadmium (Cd), a heavy metal with increasingly growing toxicological importance that is widely diffused, since it can be found in food, cigarette smoke, mines, phosphate fertilizers, and nickel-cadmium batteries (18). Cd enters the body through the gastrointestinal and the respiratory apparatus (18). Cd causes hypothyroidism (3, 19–23) and evident changes in thyroid follicles, such as decreased follicular area (24, 25), structural and ultrastructural changes of thyrocytes (3, 24, 25), increase of the interstitial connective tissue (24), and upregulation of MCD1 and CXCL10 chemokines (24).

As to the effects of Cd on C cells, few and contrasting data are present in the literature. Indeed, the chronic administration of Cd in adult rats induced proliferation of C cells (26, 27), a finding that parallels the C cell diffuse hyperplasia, hypertrophy, and hypergranulation observed in rats (3). In contrast, the acute exposure of rats to Cd, alone or co-administered with ethanol, induced weakening of CT immunoreactivity (28, 29). As far as we know, no data are currently available on the effects of Cd on murine C cells and on their possible protection by exogenous substances. Recently, our group demonstrated that mouse thyrocytes are protected from the toxic action of Cd when Cd is co-administred with a combination of myo-inositol (MI) and selenium (Se), such protection being greater than that conferred by the co-administration of either MI alone or Se alone (24).

On the basis of this background, and taking into account that: (i) C cells interact with follicular cells by being hyperplastic when TSH is high (30); (ii) no objective morphometric analysis has been conducted in previous studies on Cd-induced C cells damage (3, 26, 28, 29); (iii) no data are available regarding the effects of MI alone, Se alone and the association of MI with Se on the Cd-induced C cells damage; and (iv) many naturally occurring nutraceuticals, including MI and Se, are being increasingly used in the clinical pratice (31), we intended to demonstrate the protecting role of Se and MI on the murine C cells of thyroid gland exposed to Cd. Their protective effects were compared with those by resveratrol, another naturally anti-oxidant with beneficial effects (31–33) that had not been tested in our previous study on Cd-induced toxicity in testis (34) and kidney (35).

## Materials and Methods

### Drugs and Chemicals

CdCl_2_, Se, and resveratrol were purchased from Sigma-Aldrich Srl (Milan, Italy). MI was a kind gift of LO.LI. Pharma S.r.l. (Rome, Italy). All chemicals not otherwise mentioned were commercially available reagent grade.

### Experimental Protocol

The standards for care and use of animals as per guidelines issued by the Animal Research Reporting *In Vivo* Experiments (ARRIVE) were followed in the present study; all procedures were evaluated and approved by the Italian Health Ministry (project identification code: 112/2017 - PR).

Ninety-eight male C57 BL/6J mice (25–30g; age 8–10 weeks) were obtained from Charles River Laboratories Italia Srl (Calco, LC, Italy) and stored at the animal house Faculty of our University hospital. The animals were provided a standard diet ad libitum and had free approach to water; they were kept on a 12-h light/dark cycle. They were randomly divided into 14 groups of 7 mice each. Seven groups were indicated as controls, namely, (i) 0.9% NaCl (vehicle; 1 ml/kg); (ii) seleno-L-methionine (Se, 0.2 mg/kg); (iii) Se (0.4 mg/kg); (iv) MI (360 mg/kg); (v) MI (360 mg/kg) plus Se (0.2 mg/kg); (vi) MI (360 mg/kg) plus Se (0.4 mg/kg)]; (vii) resveratrol (20 mg/kg). The remaining seven groups were challenged as follows: (viii) CdCl_2_ (2 mg/kg) plus vehicle; (ix) CdCl_2_ (2 mg/kg) plus Se (0.2 mg/kg); (x) CdCl_2_ (2 mg/kg) plus Se (0.4 mg/kg); (xi) CdCl_2_ (2 mg/kg) plus MI (360 mg/kg); (xii) CdCl_2_ (2 mg/kg) plus MI (360 mg/kg) plus Se (0.2 mg/kg); (xiii) CdCl_2_ (2 mg/kg) plus MI (360 mg/kg) plus Se (0.4 mg/kg); (xiv) CdCl_2_ (2 mg/kg) plus resveratrol (20 mg/kg) (24, 36, 37). Resveratrol was tested in our previous study on the follicles (24), where it turned out to exert a protection equivalent to that of MI alone. These data prompted us to verify in new experiment such equipotency of resveratrol and MI. CdCl_2_ and NaCl were administered intraperitoneally (i.p.), while MI, Se and resveratrol per os. MI was ready for use, CdCl_2_ and Se were diluted in 0.9% NaCl, while resveratrol was dispersed in 0.5% methylcellulose. The basis for using the said doses of nutraceuticals was specified in previous works by our group (24, 34–36) and other authors (37, 38). After 14 days of treatment, mice were sacrificed with an overdose of ketamine and xylazine, and the thyroids were collected and processed for histological, immunohistochemical, and morphometric procedures. Within the budget limits of this unfunded research, we could not afford elaborated mechanistic investigations on the C cell protection from Cd effects exerted by the nutraceuticals tested. However, in view of the proliferative effects on the C cells exerted by Cd in rodents (see *Introduction*) and since the only disorders of clinical relevance associated with C cells are proliferative in nature [C cell hyperplasia and medullary thyroid cancer (MTC)], the fundamental investigation to be performed had to be morphological. Also, based on these considerations, it was logical to consider promotion of apoptosis as a mechanism for the expected anti-hyperplastic effects given by the nutraceuticals we tested.

### Histological Evaluation

The thyroid glands were fixed in 4% paraformaldehyde in 0.2 M phosphate buffer saline (PBS), dehydrated in graded ethanol, cleared in xylene, and embedded in paraffin (Paraplast, SPI Supplies, West Chester, PA, USA). Paraffin blocks were cut in a rotary microtome (RM2125 RT, Leica Instruments, Nussloch, Germany), and 5-μm sections, all obtained from the middle portions of the thyroid lobes, were cleared with xylene, rehydrated in graded ethanol and treated with HE stain (39). The images were taken with a Nikon Ci-L (Nikon Instruments, Tokyo, Japan) light microscope fitted with a digital camera Nikon Ds-Ri2 and saved as Tagged Image Format Files (TIFF) using the Adobe Photoshop CS5 12.1 software. For parameters evaluated on HE-stained sections, see *Morphometric Evaluation*.

### Immunohistochemistry for CT

From the same specimens used for histological evaluation, paraffin-embedded 5-μm sections were mounted on Polysine slides (Thermo Fisher Scientific, Waltham, MA, USA), cleared in xylene, and rehydrated in decreasing concentrations of ethanol. Antigen retrieval was performed with pH 6.0 buffer citrate and endogenous peroxidase was blocked with 0.3% H_2_O_2_ in PBS. Primary antibody (calcitonin, 1:100, Ventana Medical Systems, Tucson, AZ, USA) was incubated overnight at 4°C in a moisturized chamber and the day after the secondary antibody (anti-mouse, Vectastain, Vector, Burlingame, CA, USA) was added and the reaction was visualized with 3,3′-Diaminobenzidine (DAB) (Sigma-Aldrich, Milan, Italy). Counterstaining was performed in Mayer’s haematoxylin. Appropriate positive and negative controls were used in each test. Slides were photographed with a Nikon Ci-L light microscope using a digital camera Nikon Ds-Ri2. For parameters evaluated on CT-stained cells, see *Morphometric Evaluation*.

### Evaluation of Apoptosis With Terminal Deoxynucleotidyl Transferase dUTP Nick End Labeling (TUNEL) Assay

An apoptosis detection kit (*In situ* Apoptosis Detection kit, Abcam, Cambridge, UK) was used for the TUNEL technique following the manufacturer’s instructions. In brief, from the same specimens used for histological evaluation, 5-μm sections were cleared in xylene and rehydrated in ethanol. After permeabilization with proteinase K, endogenous peroxidase activity was blocked with 3% H_2_O_2_ in methanol. Sections were incubated with terminal deoxynucleotidyl transferase, with biotin-labeled deoxynucleotides, with streptavidin-horseradish peroxidase conjugate, and lastly with the diaminobenzidine solution. Counterstaining was performed in Mayer’s haematoxylin. The slides were photographed with a Nikon Ci-L light microscope using a digital camera Nikon Ds-Ri2. For evaluation of the distribution of the apoptotic C cells, see *Morphometric Evaluation*.

### Morphometric Evaluation

All micrographs were printed at the same final magnification (800×) and were assessed by two trained observers who were blinded to the experimental group of mice. To confirm the different protective action of Se, MI, and resveratrol on thyroid follicles, we evaluated the mean follicular diameter, which, in the previous paper (24), was already taken into account to calculate the radius of the follicle and, therefore, the follicular area. In particular, the smallest and largest diameters of each follicle were measured with the ImageJ software (http://rsb.info.nih.gov/ij/; freely available from the National Institutes of Health, Bethesda, MD, USA) using the function “analyze > measure”. The sum of the smallest and largest diameters of each follicle, expressed in μm, was divided by two. The mean diameter of 20 follicles was measured in each thyroid gland.

For the evaluation of the immunoreactivity for CT, the number of positive cells was counted from 10 nonserial sections of each group of mice, selecting two unit areas (UA) of 0.1 μm^2^ (316 μm × 316 μm). From the same UA, the C cells area and their cytoplasmatic density were calculated with the said public domain ImageJ software, using the function “analyze > measure”. Only C cells with well evident nuclei were chosen: the area of the cells was expressed in µm^2^ and the cytoplasmatic density was calculated in optical units (OU). The scale for OU is comprised between 0 and 255, with from 0 indicating the highest intensity (black) and 255 indicating the lowest intensity (white). For this reason, in the appropriate figure, in the vertical axis, the position of 0 and 255 had to be reversed, with 0 on the top and 255 at the bottom. The nucleus was not included in the calculation of the density.

In order to establish the distribution of apoptosis, the mean number of TUNEL-positive C cells per UA of 0.1 μm^2^ (316 μm × 316 μm) was calculated from 10 nonserial sections of each group. Cells overlapping the right and the top boundaries of the areas were not included in the evaluation, while the cells overlapping the left and the bottom boundaries were counted.

### Statistical Analysis

Values are expressed as mean ± standard error (SE). The statistical significances of differences among all experimental groups for a given parameter having Gaussian distribution were handled by ANOVA. A post-hoc analysis between any two experimental groups was handled with the Students’ t-test using the Bonferroni correction to account for multiple comparisons. A P value of ≤ 0.05 was considered statistically significant.

## Results

We will describe first data for the seven groups of the mice that received no Cd, and that we refer to as control mice. These control mice are those treated for 14 days with (i) vehicle, (ii) 0.2 mg/kg/day Se, (iii) 0.4 mg/kg/day Se, (iv) MI, (v) MI plus 0.2 mg/kg/day Se, (vi) MI plus 0.4 mg/kg Se, and (vii) 20 mg/kg resveratrol. Therefore, for sake of simplification, control data are pooled, and in all figures of this paper they are presented as a single image (panel A) and a single mean ± SE (either bar A or circle A).

### Histological Evaluation of Se and MI Effects on Follicular Diameter

**Figure 1:**
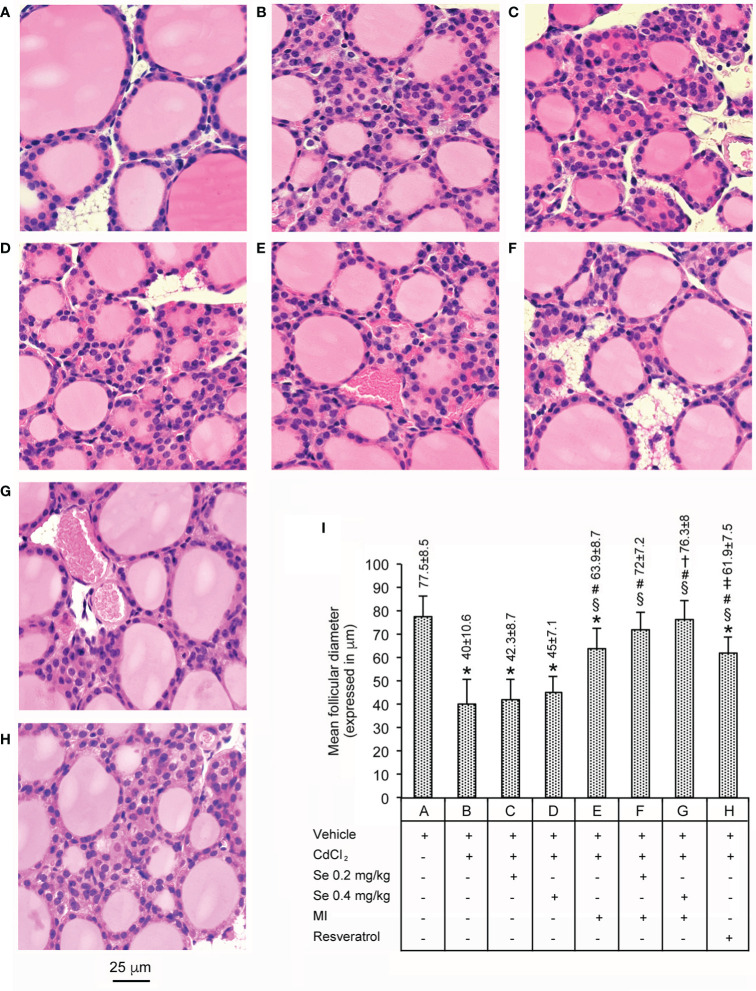
Histological organization of the thyroid (Hematoxylin-Eosin stain - Scale bar: 25 µm) in different groups of adult male mice (7 mice/group). Bars A through H in the graph of panel I quantify (mean ± SE) what is shown in the corresponding images of panels. **(A)** through **(H)**. Panel and bar **(A)**: Not to complicate the figure, the image and the mean ± SE of only one of the seven controls (administration of vehicle alone) are provided, since they overlapped images and means ± SE of the remaining six controls (0.2 mg/kg/day Se alone; 0.4 mg/kg/day Se alone; MI alone; MI plus 0.2 mg/kg/day Se; MI plus 0.4 mg/kg Se; resveratrol). Control mice have normal thyroid structure. Panel and bar **(B)**: CdCl_2_-treated mice show a significantly lower mean follicular diameter. Panels and bars **(C, D)**: In mice treated with CdCl_2_ plus Se 0.2 mg/kg or CdCl_2_ plus Se 0.4 mg/kg, the mean follicular diameter is still significantly low when compared to controls. Panel and bar **(E)**: In mice treated with CdCl_2_ plus MI, the mean follicular diameter increases, even if it is significantly lower than controls. Panels and bars **(F, G)**: In mice treated with CdCl_2_ plus MI and Se 0.2 mg/kg or CdCl_2_ plus MI and Se 0.4 mg/kg, the mean follicular diameter is similar to controls. Panel and bar **(H)**: In mice treated with CdCl_2_ plus resveratrol, the mean follicular diameter was significantly higher than CdCl_2_ alone treated mice, but significantly lower than CdCl_2_ plus MI and Se 0.2 mg/kg/day, CdCl_2_ plus MI and Se 0.4 mg/kg/day, and controls mice. Panel **(I)**: Mean values ± standard error of follicular diameter in the different groups of mice. Symbols for statistics: *P < 0.05 versus control; ^§^P < 0.05 versus CdCl_2_ plus vehicle; ^#^P < 0.05 versus CdCl_2_ plus Se 0.2 or 0.4 mg/kg; ^†^P < 0.05 versus CdCl_2_ plus MI; ^‡^P < 0.05 versus CdCl_2_ plus MI plus 0.2 mg/kg Se and CdCl_2_ plus MI plus 0.4 mg/kg Se.

These data are shown in **Figure 1**. All seven control groups of animals showed thyroids with normal morphology, confirming our previous observations (24). Therefore, for the clarity of images a single datum is provided as representative of all controls (**Figure 1A**, and bar A in panel I). The thyroids of mice challenged with CdCl_2_ (panel and bar B), had a significantly lower mean follicular diameter (−48% compared with controls). In mice treated with CdCl_2_ plus Se 0.2 mg/kg/day or CdCl_2_ plus Se 0.4 mg/kg/day, the mean follicular diameters were also significantly lower when compared to controls [−43% (panel and bar C) and −41% (panel and bar D), respectively], but comparable to the said −48% of mice treated with CdCl_2_ alone. In mice treated with CdCl_2_ plus MI (panel and bar E), the mean follicular diameter increased, even if it was significantly lower than controls (−17.5%) and higher than CdCl_2_ alone treated mice (+37.5%). In mice treated with CdCl_2_ plus MI and Se 0.2 mg/kg/day (panel and bar F) or CdCl_2_ plus MI and Se 0.4 mg/kg/day (panel and bar G), the mean follicular diameter was similar to controls (−7% and −1.5%, respectively) and significantly higher than CdCl_2_ alone treated mice (+55.5% and +57.6% respectively). In mice treated with CdCl_2_ plus resveratrol (panel and bar H), the mean follicular diameter was significantly higher than CdCl_2_ alone treated mice (+35.4%), but significantly lower than CdCl_2_ plus MI and Se 0.2 mg/kg/day (−14.1%), CdCl_2_ plus MI and Se 0.4 mg/kg/day (−18.9%) and controls mice (−20.2%).

### Immunohistochemistry for CT

**Figure 2:**
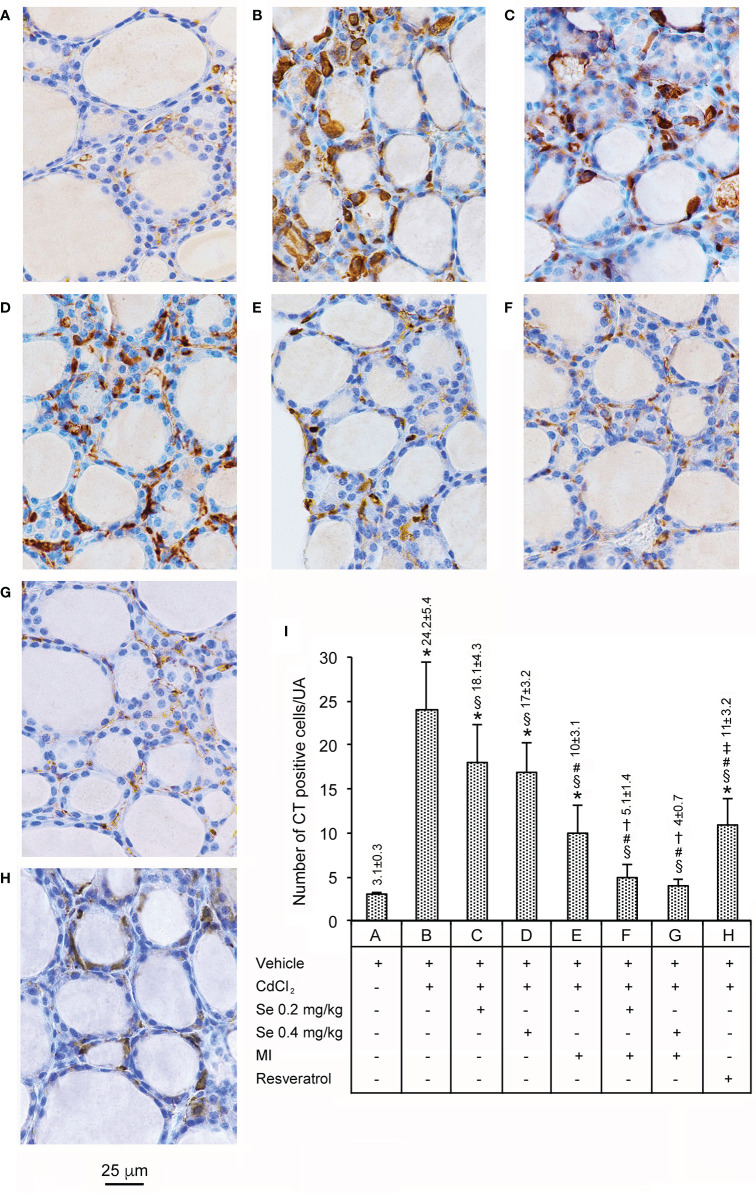
Immunohistochemical expression of CT in the thyroid (Scale bar: 25 µm) in different groups of adult male mice (7 mice/group). Panels and bars are as in **Figure 1**. Panel and bar **(A)**: In controls, only few cells show CT immunoreactivity. Panel and bar **(B)**: CdCl_2_-treated mice displayed a marked increase of cytoplasmic CT immunoreactivity. Panels and bars **(C, D)**: In CdCl_2_ plus Se 0.2 mg/kg/day and CdCl_2_ plus Se 0.4 mg/kg-treated mice, the number of CT positive cells is significantly decreased, but still higher than controls. Panel and bar **(E)**: In CdCl_2_ plus MI-treated mice, CT positive cells are significantly fewer and show reduced cytoplasmic stain. Panels and bars **(F, G)**: In the thyroid of CdCl_2_ plus MI and Se at 0.2 or 0.4 mg/kg treated mice, CT immunoreactivity is significantly reduced. Panel and bar **(H)**: In mice treated with CdCl_2_ plus resveratrol, the number of CT positive cells is significantly decreased, but still higher than controls. Panel **(I)**: Mean number ± standard error of CT positive cells/UA in the different groups of mice. Symbols for statistics ^*^P < 0.05 versus control; ^§^P < 0.05 versus CdCl_2_ plus vehicle; ^#^P < 0.05 versus CdCl_2_ plus Se 0.2 and 0.4 mg/kg; ^†^P < 0.05 versus CdCl_2_ plus MI; ^‡^P < 0.05 versus CdCl_2_ plus MI plus 0.2 mg/kg Se and CdCl_2_ plus MI plus 0.4 mg/kg Se.

Data are presented in **Figure 2**. In all seven control groups of animals, CT immunoreactivity was limited only to a few cells (panel A, and bar A in panel I). CdCl_2_ plus vehicle treated mice displayed a 7.8-fold increase in the number of CT-positive cells when compared to controls (panel and bar B). In the thyroid of CdCl_2_ plus Se 0.2 (panel and bar C) and CdCl_2_ plus Se 0.4 mg/kg/day treated mice 2 (panel and bar D), the number of CT positive cells was significantly decreased (1.3 and 1.4-fold, respectively), when compared to CdCl_2_ + vehicle, but their number was still high compared with controls (5.8- and 5.5-fold, respectively). In mice treated with CdCl_2_ plus MI (panel and bar E), CT positive cells were significantly fewer (2.4-fold compared to CdCl_2_ + vehicle) but, again, still more numerous compared with controls (3.2 fold). In the thyroid of CdCl_2_ plus MI and Se at 0.2 mg/kg/day (panel and bar F) or CdCl_2_ plus MI and Se at 0.4 mg/kg/day treated mice (panel and bar G), the number of CT positive cells was significantly reduced compared to CdCl_2_ + vehicle (4.7- and 6-fold, respectively), but insignificantly reduced compared to controls (only 1.6- and 1.3-fold, respectively). In mice treated with CdCl_2_ plus resveratrol (panel and bar H), CT positive cells were significantly fewer than CdCl_2_ alone treated mice (2.2-fold), but significantly more numerous than controls (3.5-fold), CdCl_2_ plus MI and Se 0.2 mg/kg/day (2.1-fold), and CdCl_2_ plus MI and Se 0.4 mg/kg/day treated mice (2.7-fold).

### Morphometric Analysis for CT

**Figure 3:**
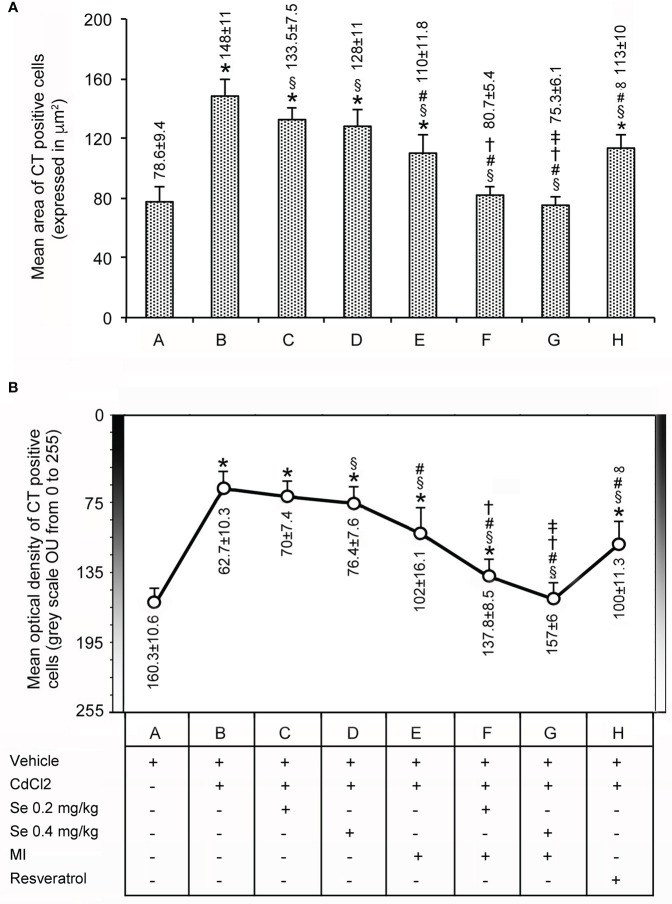
Panel **(A)**: Mean area ± standard error (expressed in µm^2^) of CT positive C cells in the thyroid of the same groups of mice as in **Figure 1**. Panel **(B)**: Mean optical density (expressed in OU from 0 indicating the highest intensity to 255 indicating the lowest intensity) ± standard error of CT positive C cells in the thyroid from the same groups of mice as above. Symbols for statistics *P < 0.05 versus control; ^§^P < 0.05 versus CdCl_2_ plus vehicle; ^#^P <0.05 versus CdCl_2_ plus Se 0.2 and 0.4 mg/kg; ^†^P < 0.05 versus CdCl_2_ plus MI; ^‡^P <0.05 versus CdCl_2_ plus Se 0.2 mg/kg plus MI;^ ∞^P < 0.05 versus CdCl_2_ plus MI plus 0.2 mg/kg Se and CdCl_2_ plus MI plus 0.4 mg/kg Se.

Data are shown in **Figure 3**. In the seven control groups, the mean area (top panel) and the optical density (bottom panel) of the CT-positive cells averaged 78.6 ± 9.4 µm^2^ (bar A in the top panel) and 160.3 ± 10.6 OU (circle A in the bottom panel), respectively. In mice treated with CdCl_2_ plus vehicle (bar and circle B), both parameters were significantly increased compared to controls, the mean area being nearly 2-fold higher and the optical density 2.5-fold higher. In the thyroid of CdCl_2_ plus Se 0.2 mg/kg/day (bar and circle C) and CdCl_2_ plus Se 0.4 mg/kg/day treated mice (bar and circle D), there was a mild, but significant decrease of both values as compared to CdCl_2_ + vehicle. The decrease was more evident in mice treated with CdCl_2_ plus MI (bar and circle E), since the mean area was only 1.6-fold higher than controls and the mean optical density was 1.6-fold higher than controls. In this group of mice, both parameters were significantly lower than those of CdCl_2_ + vehicle treated mice (1.3-fold). In the thyroid of CdCl_2_ plus MI and Se at 0.2 mg/kg/day (bar and circle F) or CdCl_2_ plus Se at 0.4 mg/kg/day treated mice (bar and circle G), both parameters were even more significantly reduced compared to CdCl_2_ + vehicle, and dose-dependently since in CdCl_2_ plus MI and Se at 0.4 mg/kg/day treated mice the greater dose of Se was associated with values significantly lower than in CdCl_2_ plus MI and Se at 0.2 mg/kg/day treated mice. In mice treated with CdCl_2_ plus resveratrol (bar and circle H), both the mean area and the mean optical density of the CT-positive cells was significantly reduced if compared to CdCl_2_ alone treated mice (1.3- and 1.6-fold, respectively).

### Measurement of Apoptosis With TUNEL Assay

**Figure 4:**
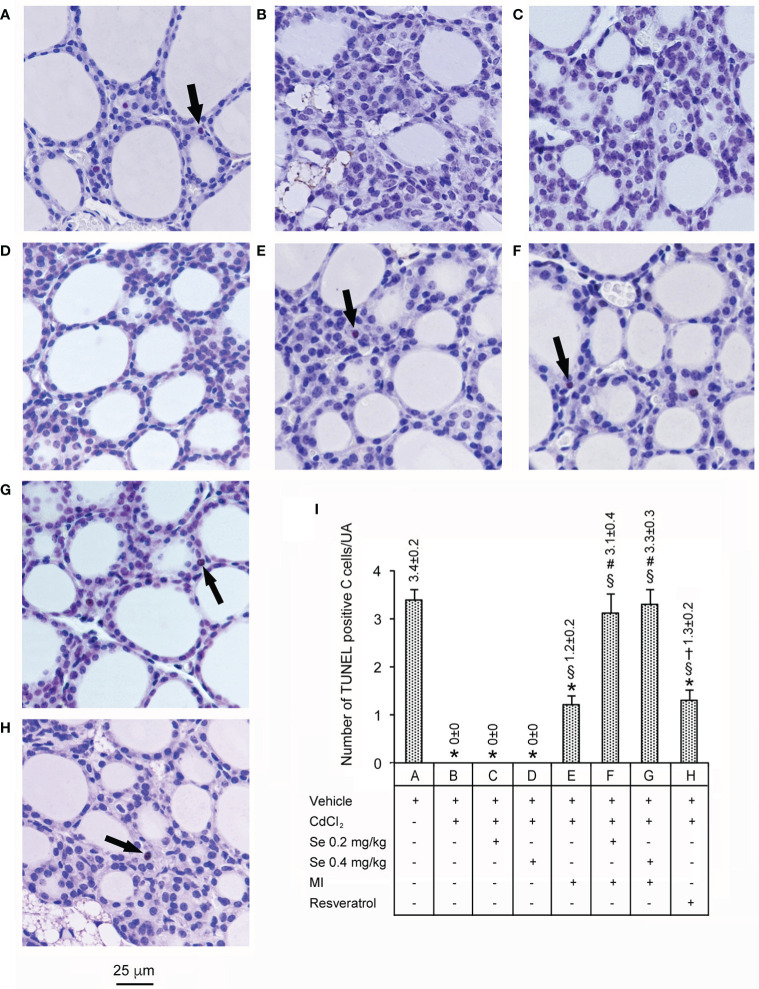
Assessment of apoptosis with the TUNEL staining technique in the thyroid of the same groups of mice as in **Figure 1**. (Scale bar: 25 µm). Panel and bar **(A)**: In controls, few cells show TUNEL positive reaction (arrow). Panels and bars **(B–D)**: In CdCl_2_ plus vehicle, CdCl_2_ plus Se 0.2 mg/kg/day and CdCl_2_ plus Se 0.4 mg/kg/day treated mice no TUNEL positive cells are present. Panel and bar **(E)**: In mice treated with CdCl_2_ plus MI only isolated TUNEL positive cells (arrow) are evident. Panels and bars **(F, G)**: In the thyroid of mice treated with CdCl_2_ plus MI plus Se at 0.2 mg/kg/day and CdCl_2_ plus MI plus Se at 0.4 mg/kg/day TUNEL positive cells (arrow) show values comparable to controls. Panel and bar **(H)**: In mice treated with CdCl_2_ plus resveratrol, isolated TUNEL positive cells (arrow) are present. Panel **(I)**: Mean number ± standard error of TUNEL positive cells/unit of area (UA) in the different groups of mice. Symbols for statistics: *P < 0.05 versus control; ^§^P < 0.05 versus CdCl_2_ plus vehicle, CdCl_2_ plus Se 0.2 and 0.4 mg/kg; ^#^P < 0.05 versus CdCl_2_ plus MI; ^†^P < 0.05 versus CdCl_2_ plus MI plus 0.2 mg/kg Se and CdCl_2_ plus MI plus 0.4 mg/kg Se.

Data are illustrated in **Figure 4**. In control mice (panel and bar A), few C cells showed TUNEL positive reaction. In contrast, in CdCl_2_ plus vehicle (panel and bar B), no such cells were evident, a change that was not prevented by CdCl_2_ plus Se 0.2 mg/kg/day and CdCl_2_ plus Se 0.4 mg/kg/day (panel and bar C, and panel and bar D, respectively). In mice treated with CdCl_2_ plus MI alone (panel and bar E), isolated TUNEL positive C cells became evident again, but the number was approximately 3 times lower than controls. In the thyroid of mice treated with CdCl_2_ plus MI plus Se at 0.2 mg/kg/day (panel and bar F) and CdCl_2_ plus MI plus Se 0.4 mg/kg/day (panel and bar G) TUNEL positive C cells show values comparable to controls, with a trendwise dose-dependent effect. In mice treated with CdCl_2_ plus resveratrol (panel and bar H), isolated TUNEL positive C cells were present, but their number was 2.6 times lower than controls.

## Discussion

As mentioned in the Introduction, Cd is naturally occurring heavy metal with increasingly growing toxicological importance. Various studies showed the effects of this metal on hormones (21, 40, 41): in particular, thyroid disruption was demonstrated in humans and animal models both *in vivo* and *in vitro* (also see *Introduction*). In humans, Cd exposure induces thyroid dysfunction and autoimmunity, based on the increased serum levels of TSH (42) and thyroid autoantibodies (43). In animal models (rats), Cd increased thyroid gland weight and serum TSH concentration (3, 19, 20). As to the morphological aspects, Cd increased the height of follicular cells, decreased the area of the follicles (24, 25), and induced an infiltration by mononuclear cells (44, 45). No convincing data are available on the role of Cd on apoptosis in the thyroid (25), as no structural evaluation was performed. The said previous data of Cd-induced decreased follicular area (24) were confirmed now by the demonstration of Cd-induced decrease in follicle mean diameter. In order to evaluate further elements of thyroid behavior after Cd-challenge, we examined the other thyroid cell population, which is the C cells, whose embryological origin, physiological role and morphology are different from the already examined thyrocytes.

As to C cells, they represent the CT-synthesizing cells. C cells are difficult to identify in sections stained with H&E. Therefore, the most valid procedure for their identification implicates the use of immunohistochemical techniques with antibodies against CT (3, 46). C cells can be located between follicular cells, but separated from the colloid, or externally to the follicular epithelium; their nuclei are somewhat larger and paler compared to follicular cells (46).

As to the modifications of C cells after Cd challenge, data in the literature are few and apparently contrasting. In fact, chronic administration of Cd in adult rats triggered hyperplasia and hypertrophy of CT-positive C cells, their cytoplasm showing evident hypergranulation (3, 26, 27). In contrast, acute exposure to Cd, alone or coadministered with ethanol, induced weakening of CT immunohistochemical reaction, which was considered the result of an intracellular depauperation subsequent to the increased CT secretion (28, 29). However, at a histopathology level, all previous authors based their results on the effects of Cd challenge only on subjective appraisal of C cells morphology and intensity of CT immunohistochemical reaction, thus omitting a morphometric analysis with objective histopathological scores. As also suggested by Martin-Lacave et al (30)., we utilized the powerful technique of morphometric analysis with the ImageJ software, thus enabling us to provide, the first detailed analysis of C cells histopathology in Cd-challenged animals. By doing so, in addition to the previously published follicular changes (24) indicated by the significant reduction of their mean diameter, we have demonstrated hyperplastic and hypertrophic changes of C cells.

Even if hypertrophy indicates an increase in cell size and hyperplasia indicates an increase in cell number, they frequently coexist, because they are triggered by the same stimulus such as hormones and/or growth factors (47). Since it was demonstrated that primary hypothyroidism with its associated increase of serum levels of TSH induces C cells hyperplasia (such hyperplasia being reversed by T4 administration) (30), the changes in number and size of C cells that we observed after Cd challenge could be related to the Cd-induced hypothyroidism (3, 19–21). Indeed, TSH receptors were detected in C cells (30).

We now discuss the effects of natural antioxidants in Cd-challenged mice. Se decreases the tissue burden induced by heavy metals, including Cd (48), through different mechanisms. Acute toxicity studies have demonstrated that, after simultaneous administration of Se and Cd, tissue levels of both metals increased, but Cd toxicity decreased, owing to the inert nature of the Cd/Se complex (49). Furthermore, a protection from Cd toxicity could be related to the action of Se-dependent antioxidant enzymes, thus reducing the oxidative stress and counteracting the apoptosis induced by the endoplasmic reticulum stress (50). As we report in the present paper, the histopathological evaluation of the C cells (number, area, and cytoplasmic density) and the apoptotic cells number after treatment with Se at either 0.2 or 0.4 mg/kg/day failed to demonstrate a significant protection from Cd, as all the considered parameters were mildly and insignificantly modified.

As to MI, this carbocyclic sugar is involved in various cellular processes, as it lowers the oxidative stress by stimulating the natural antioxidant defenses, increasing superoxide dismutase (SOD), catalase (CAT), and glutathione (GSH) levels (51), with favorable repercussions even in the setting of thyroid autoimmunity (52–54). In our study, the morphological and morphometric evaluation of number, area and cytoplasmic density of the C cells, and the number of apoptotic cells after treatment with MI showed a significant shielding from Cd-induced damage, even if none of the considered parameters were superimposable to Cd-unexposed controls. For all parameters tested, the protection of MI was comparable to that observed with resveratrol.

When the combined action of Se and MI was tested, we found that the protection conferred by MI alone was amplified, as indicated by the marked improvement of all parameters. Indeed, we confirmed the positive role on the follicles, as indicated by the increase in their mean diameter, and we demonstrated a reduction of the hyperplastic and hypertrophic changes of the C cells, a decrease of their cytoplasmic density and an increase in the number of TUNEL positive cells, indicating the restoration of normal apoptotic activity (23).

Limitations of our study are the lack of biochemical investigations for the protection of the C cells from the Cd-induced hyperplasia and hypertrophy. However, because the clinically relevant disorders associated with C cells are proliferative in nature (C cells hyperplasia and MTC), though MTC results from mutations of certain oncogenes as opposed to Cd exposure, the fundamental investigations had to be morphological. We are tempted to speculate that especially the most effective nutraceutical (that is, the association of MI + Se 0.4 mg/kg) is worthy of being tested for antiproliferative action on cultures of MTC cell lines. The translational application would be the possible prevention, retardation, and/or loss of aggressive behavior of malignancy in consanguineous of patients with familial MTC.

Strengths of our work are having replicated our previous data (24) on protection of the follicular epithelium and, particularly, the similar potency of MI and resveratrol at the doses tested. Another strength is having expanded the spectrum of cell types protected by nutraceuticals, and consistently with the greatest benefit conferred by the association MI + Se 0.4 mg/kg. Thus, C cells add to the list composed by thyrocytes (24), spermatozoa and Leydig cells (34), and glomerular and tubular cells (35). A final strength is the following. We paid particular attention to the morphological evaluation of the C cells. In fact, since the number of C cells is known to increase with age of the animals (55), we evaluated mice between the first 20 days after birth and the first year of age. Furthermore, in order to avoid any bias correlated to the site of analysis of the specimens, all sections were obtained from the middle portions of the thyroid lobes, where C cells of rodents abound in basal conditions (7–9).

Indeed, for the biochemical parameters associated with testing anti-oxidant activity (GSH, MDA, iNOS, TNF-α) in testes (34), the hierarchy of protection from Cd effects (MI + Se 0.4 mg/kg > MI + Se 0.2 mg/kg > MI > Se 0.4 mg/kg > Se 0.2 mg/kg) paralleled the hierarchy observed for morphological parameters in testes (34). The same hierarchy based on morphology, held for the two cells population of thyroid: thyrocytes (24 and present manuscript) and parafollicular cells (present manuscript).

In conclusion, when the data presented here are taken together with the maximal protection conferred by the said combination of MI and Se from Cd-induced testicular damage effect, then this combination might protect multiple endocrine districts. This warrants testing of other endocrine disruptors as well as clinical trials on occupational exposure to cadmium.

## Data Availability Statement

The raw data supporting the conclusions of this article will be made available by the authors, without undue reservation.

## Ethics Statement

The animal study was reviewed and approved by Italian Health Ministry [project identification code: 112/201 Italian Health Ministry (project identification code: 112/2017-PR).7-PR].

## Author Contributions

SB, AM, and GPa designed the study. DP, AI, LM, NI, and GPi collected and analyzed the data. AA, PF, and FS provided intellectual input. LM, DP, and SB drafted and wrote the manuscript. SB, AA, AM, and GPa performed critical revisions to the manuscript. All authors contributed to the article and approved the submitted version.

## Funding

The work has been supported by Departmental funding of LM. Department of Clinical and Experimental Medicine, University of Messina.

## Conflict of Interest

SB has been an invited speaker for Lo.Li. Pharma, which provided us with pure MI, but had no role in the design of the study; in the collection, analyses, or interpretation of data; in the writing of the manuscript; or in the decision to publish the results.

The remaining authors declare that the research was conducted in the absence of any commercial or financial relationships that could be construed as a potential conflict of interest.

The reviewer SMF declared a shared affiliation with the authors AA and PF to the handling editor at time of review.
